# High Thyroid-Stimulating Hormone and Low Free Triiodothyronine Levels Are Associated with Chronic Kidney Disease in Three Population-Based Studies from Germany

**DOI:** 10.3390/jcm12175763

**Published:** 2023-09-04

**Authors:** Till Ittermann, Sabrina von Rheinbaben, Marcello R. P. Markus, Marcus Dörr, Antje Steveling, Matthias Nauck, Alexander Teumer, Maik Gollasch, Dominik Spira, Maximilian König, Ilja Demuth, Elisabeth Steinhagen-Thiessen, Henry Völzke, Sylvia Stracke

**Affiliations:** 1 Institute for Community Medicine—SHIP Clinical-Epidemiological Research, University Medicine Greifswald, 17475 Greifswald, Germany; voelzke@uni-greifswald.de; 2 Department of Medicine A—Gastroenterology, Nephrology, Endocrinology and Rheumatology, University Medicine Greifswald, 17475 Greifswald, Germany; sabrina.freiinvonrheinbaben@med.uni-greifswald.de (S.v.R.); antje.steveling@med.uni-greifswald.de (A.S.); sylvia.stracke@med.uni-greifswald.de (S.S.); 3 Department of Internal Medicine B—Cardiology, Intensive Care, Pulmonary Medicine and Infectious Diseases, University Medicine Greifswald, 17475 Greifswald, Germany; marcello.markus@uni-greifswald.de (M.R.P.M.); mdoerr@uni-greifswald.de (M.D.); 4 DZHK (German Center for Cardiovascular Research), Partner Site Greifswald, 17475 Greifswald, Germany; matthias.nauck@med.uni-greifswald.de (M.N.); ateumer@uni-greifswald.de (A.T.); 5 Institute of Clinical Chemistry and Laboratory Medicine, University Medicine Greifswald, 17475 Greifswald, Germany; 6 Department of Psychiatry and Psychotherapy, University Medicine Greifswald, 17475 Greifswald, Germany; 7 Department of Internal Medicine and Geriatrics, University Medicine Greifswald, 17475 Greifswald, Germany; maik.gollasch@med.uni-greifswald.de (M.G.); maximilian.koenig@med.uni-greifswald.de (M.K.); 8 Department of Endocrinology and Metabolism, Charité—Universitätsmedizin Berlin, Corporate Member of Freie Universität Berlin, Humboldt-Universität zu Berlin, and Berlin Institute of Health, 13353 Berlin, Germany; dominik.spira@charite.de (D.S.); ilja.demuth@charite.de (I.D.); elisabeth.steinhagen-thiessen@charite.de (E.S.-T.); 9 BCRT—Berlin Institute of Health Center for Regenerative Therapies, Charité—Universitätsmedizin Berlin, 13353 Berlin, Germany

**Keywords:** thyroid-stimulating hormone, thyroid hormones, estimated glomerular filtration rate, albuminuria, chronic kidney disease

## Abstract

High serum thyroid-stimulating hormone (TSH) levels have previously been associated with a low estimated glomerular filtration rate (eGFR), but studies associating thyroid hormone levels with albuminuria revealed inconsistent results. We used cross-sectional data from 7933 individuals aged 20 to 93 years of the Berlin Aging Study II and the Study of Health in Pomerania to associate serum TSH, fT3, and fT4 levels with eGFR and albuminuria. In multivariable analyses adjusted for confounding, we found inverse non-linear associations of serum TSH levels with eGFR, while serum fT3 levels showed a positive association with eGFR. High as well as low serum fT4 levels were associated with a lower eGFR. Age but not sex modified the association between thyroid hormone levels and eGFR. The inverse associations between serum TSH levels and eGFR were strongest in the youngest age groups, while the positive associations between serum fT3 levels and eGFR were strongest in older individuals. No significant associations between thyroid hormone levels and albuminuria were found. Our results indicate that hypothyroidism might be associated with a reduced kidney function. Thyroid function might be more tightly related to the eGFR than to albuminuria in the general population.

## 1. Introduction

Chronic kidney disease (CKD) is defined as abnormalities in kidney function and structure for more than three months. The severity of CKD can be estimated using the “Kidney Disease Improving Global Outcomes” (KDIGO) definition, which uses measurements of glomerular filtration rate and albuminuria as markers for kidney damage and dysfunction [1]. Low glomerular filtration rate and albuminuria are both risk factors for cardiovascular events and mortality [2,3,4].

Previous cross-sectional studies showed associations between high serum thyroid-stimulating hormone (TSH) levels even within the reference range and CKD as defined by an estimated glomerular filtration rate (eGFR) <60 mL/min [5,6,7]. In a longitudinal individual participant data meta-analysis, however, no association between low thyroid function at baseline and changes in eGFR levels during 329,713 person-years was demonstrated. The authors stress that the previously observed cross-sectional associations may be explained by renal dysfunction causing thyroid hormone alterations and not vice versa [7]. In contrast to this, a Mendelian randomization study demonstrated associations of hypothyroidism and increased TSH with a decreased eGFR [8].

To date, there is limited knowledge of the association between thyroid function and albuminuria. In a large Japanese population of individuals, who underwent a health check-up, subclinical hypothyroidism was associated with albuminuria defined as a urinary albumin–creatinine ratio (UACR) ≥30 mg/g [9]. In a population-based study from China, free triiodothyronine (fT3) levels in serum were inversely associated with a UACR between 30 and 300 mg/g but there were no results reported for TSH or free thyroxine (fT4) levels [10]. Another study in 147 pre-diabetic adults reported a positive association of subclinical hypothyroidism with a UACR between 30 and 300 mg/g [11].

So far, no study has investigated associations of thyroid function as defined by serum levels of TSH, fT3, and fT4 with eGFR and albuminuria in parallel. Against this background, the aim of our study was to investigate such associations in three population-based German studies. The second aim of our study was to determine whether these associations are modified by age or sex.

## 2. Material and Methods

### 2.1. Study Population

The present analyses were based on data from two studies within the framework of the “Study of Health In Pomerania” (SHIP) and the Berlin Aging Study II (BASE-II).

**Figure 1:**
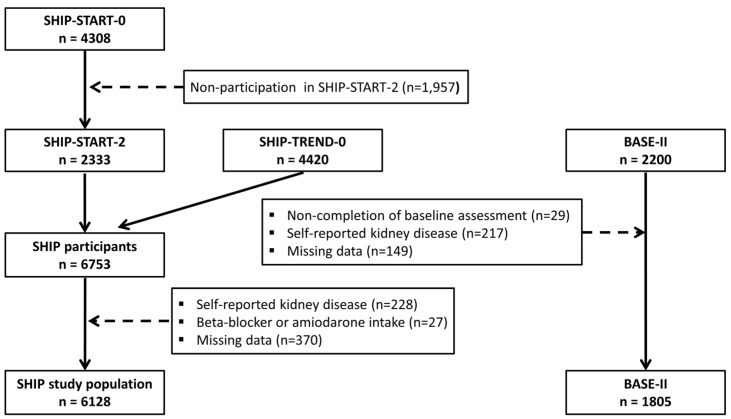
Flow chart of the study population.

The SHIP project includes several large population-based studies, which were all conducted in the northeast of Germany. In the first SHIP cohort (SHIP-START), 6265 individuals aged 20–79 years were selected from population registries, of which 4308 individuals (response 68.8%) participated between 1997 and 2001 [12]. For the present analyses, we used data from the second follow-up (SHIP-START-2), in which 2333 individuals aged 30–93 years were examined between 2008 and 2012. In parallel to SHIP-START-2, baseline examinations of a second cohort were conducted (SHIP-TREND). For SHIP-TREND, a separate stratified random sample of 8826 adults aged 20–79 years was drawn and 4420 subjects participated between 2008 and 2012 (response 50.1%). Of the 6753 individuals examined in SHIP-START-2 or SHIP-TREND-0, we excluded 228 individuals with a self-reported kidney disease, 27 taking propranolol-containing beta blockers (C07AA05) or amiodarone (C01BD01), and 370 individuals with missing data in the outcomes, exposures, or confounders. The final study population consisted of 6128 individuals (Figure 1).

The BASE-II sample comprised 2200 participants of which 2171 completed the baseline medical assessments between 2009 and 2014. The group of older participants were aged between 60 and 85 years (N = 1671), whereas the younger control group was between 22 and 37 years (N = 500) [13,14]. The participants were recruited as a convenience sample in the residents of the greater metropolitan area of Berlin, Germany. BASE-II was registered with the clinical trial registry Deutsches Register Klinischer Studien (DRKS00009277). Of the 2171 individuals examined in BASE-II, we excluded 217 individuals with a self-reported kidney disease and 149 individuals with missing data in the outcomes, exposures, or confounders. The final study population consisted of 1805 individuals (Figure 1).

### 2.2. Assessments

In SHIP, sociodemographic characteristics, type 2 diabetes, and history of kidney diseases were assessed using computer-assisted personal interviews, while in BASE-II, this information was collected from medical history recorded by a study physician. Smokers were categorized into three categories (lifetime non-smokers, former smokers, and current smokers). Medication data were obtained online using the IDOM program (online-drug-database-led medication assessment) and categorized according to the Anatomical Therapeutical Chemical (ATC) classification index. Thyroid medication was defined according to the ATC-code H03.

Blood samples were taken non-fasting in SHIP-START-2. In SHIP-Trend-0, 75% of the blood samples were taken fasting, while in BASE-II, all samples were taken fasting. All SHIP samples were analyzed in the Institute of Clinical Chemistry and Laboratory Medicine of the University Medicine Greifswald, whereas in BASE-II, laboratory parameters were determined in a commercial laboratory (Medizinisches Versorgungszentrum Labor 28 GmbH, Berlin, Germany). Low-density lipoprotein cholesterol (LDL-C), high-density lipoprotein cholesterol (HDL-C), triglycerides, and uric acid were measured photometrically in SHIP (Dimension VISTA 1500, Siemens Healthcare Diagnostics, Eschborn, Germany) and in BASE-II (Roche/Hitachi Modular instruments: ACN 435 and ACN 781 (Indianapolis, IN, USA). Serum creatinine concentrations were determined enzymatically (traceable to isotope dilution mass spectrometry; IDMS) in SHIP and through a compensated Jaffé assay, traceable to IDMS (Roche Diagnostics, Mannheim, Germany), in BASE-II. To calculate the estimated glomerular filtration rate (eGFR), we used the CKD-EPI and FAS equations [15]:

If male & creatinine ≤ 0.9: 141 × (Creatinine/0.9)^−0.411^ × 0.993Age
If male & creatinine > 0.9: 141 × (Creatinine/0.9)^−1.209^ × 0.993Age
If female & creatinine ≤ 0.7: 144 × (Creatinine/0.7)^−0.329^ × 0.993Age
If female & creatinine > 0.7: 144 × (Creatinine/0.7)^−1.209^ × 0.993Age
FAS equation [16]:
If male & age ≤ 40: 107.3/(Creatinine/0.9)
If male & age > 40: 107.3/((Creatinine/0.9) × 0.998^(Age−40)^)
If female & age ≤ 40: 107.3/(Creatinine/0.7)
If female & age > 40: 144 × ((Creatinine/0.7) × 0.998^(Age−40)^)
Low eGFR was defined as an eGFR < 60 mL/min for both definitions.

Urinary albumin excretion was expressed as the albumin/creatinine ratio (ACR) in milligram per gram. Urinary albumin concentrations were measured using nephelometric assays (SHIP: BN ProSpec Analyzer, Dade Behring, Deerfield, IL, USA; BASE: Tina-quant albumin Gen.2, Cobas C, Roche diagnostics, Indianapolis, IN, USA). Albuminuria was defined as an UACR ≥ 30 mg/g.

Serum levels of TSH, fT3, and fT4 were analyzed through homogeneous, sequential, chemiluminescent immunoassays based on LOCI^®^ technology (Dimension Vista^®^, Siemens Healthcare GmbH, Eschborn, Germany) in SHIP and through an electrochemiluminescent immunoassay (Cobas immunoassay systems, Roche diagnostics, Indianapolis, Indiana) in BASE-II. High and low TSH were defined according to the reference limits 0.49 mIU/L to 3.29 mIU/L in SHIP [17] and 0.27 mIU/L to 4.20 mIU/L in BASE-II. Subclinical hyperthyroidism was defined as low TSH in combination with fT3 and fT4 levels in the reference range. Overt hyperthyroidism was defined as low TSH in combination with increased fT3 or fT4 levels. Subclinical hypothyroidism was defined as high TSH in combination with fT3 and fT4 levels in the reference range. Overt hypothyroidism was defined as high TSH in combination with decreased fT3 or fT4 levels.

### 2.3. Statistical Methods

**Figure 2:**
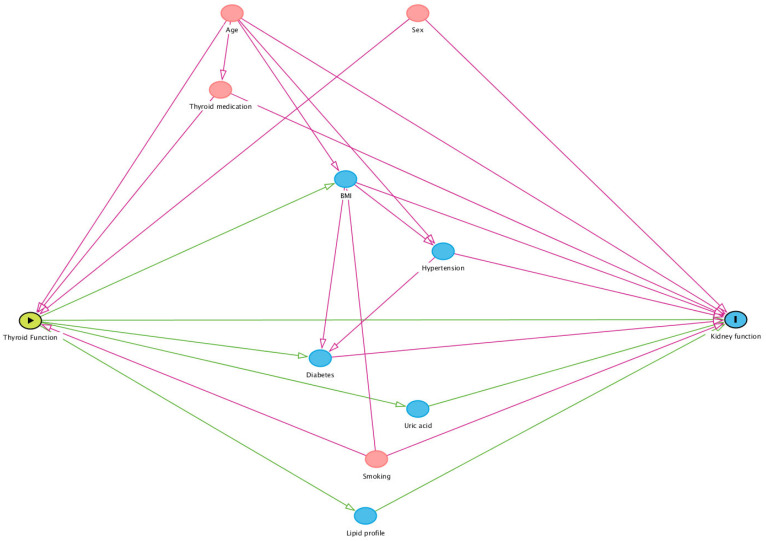
Directed acyclic graph to determine the confounding variables for the association of thyroid function with kidney function. Confounding variables are marked in red.

In groups of individuals with low TSH, high TSH, and TSH levels within the reference range, we report continuous data as median, 25th percentile, and 75th percentile, while categorical data are reported as absolute numbers and percentages. Associations of thyroid hormone levels with eGFR and albuminuria were analyzed using linear or logistic regression models adjusted for age, sex, smoking status, thyroid medication, and study. The adjustments were chosen based on a directed acyclic graph (Figure 2). For all models, potential non-linear associations were tested using fractional polynomials, a statistical method that systematically tests for transformations of the exposure [18]. A non-linear association was assumed when the model with the transformed exposure variable fitted the data significantly better than the model with the untransformed exposure variable. To investigate effect modifications by sex and age, we added interaction terms of age and sex with TSH, fT3, or fT4 to the models. Here, we also considered non-linear interactions using the mfpigen command. If a significant interaction was observed, results were visualized as marginal effects (e.g., associations of TSH with eGFR at different ages) using marginsplot in combination with the f_able command. In all analyses, *p* < 0.05 was considered as statistically significant. All analyses were conducted using Stata 17.0 (Stata Corporation, College Station, TX, USA).

## 3. Results

In the total population of all three studies, the prevalence of CKD defined as an eGFR < 60 mL/min was 7.4% for the CKD-EPI equation and 13.3% for the FAS equation. Albuminuria was observed in 976 individuals (12.3%). There were 596 individuals with low TSH levels (7.5%) and 334 individuals with high TSH levels (4.2%). In the SHIP studies, the percentage of individuals with low serum TSH levels was higher compared to BASE-II, while in BASE-II, the percentage of individuals with high TSH levels was higher than in the SHIP-studies (Table 1). Individuals with low TSH were on average older, more often hypertensive, and had a higher prevalence of CKD compared to the ones with serum TSH levels in the reference range. Individuals with high TSH were more often female and had a higher prevalence of low eGFR and albuminuria compared to individuals with serum TSH levels in the reference range.

**Figure 3:**
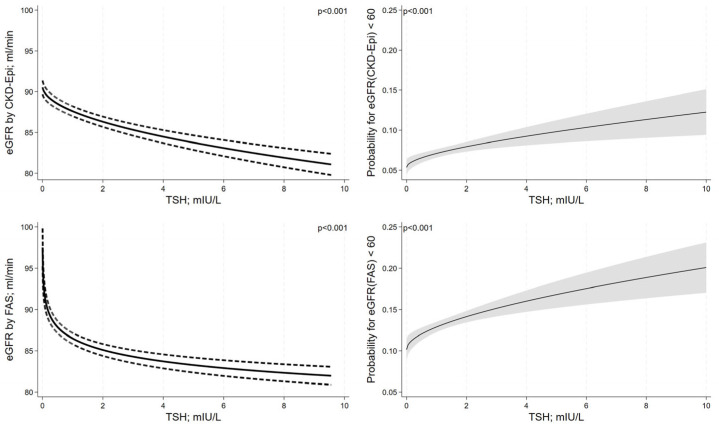
Associations between serum TSH levels and kidney function adjusted for confounding. Results are reported as adjusted regression curves (solid line) and 95% confidence interval.

After adjustment for confounding, serum TSH levels showed inverse non-linear associations (transformations: √TSH for eGFR using CKD-EPI and log(TSH) for eGFR using FAS) with the eGFR as estimated using CKD-EPI and FAS equations (Figure 3). The shape of the non-linear association between serum TSH levels and eGFR was different for the CKD-EPI and the FAS equation. For the FAS equation, we observed a more pronounced decrease in the eGFR with increasing serum TSH levels in the low range of TSH (<1 mIU/L) than for the CKD-EPI equation. Similarly, we found a significant non-linear increase in the risk for an eGFR < 60 mL/min for both equations with increasing serum TSH levels (transformation √TSH for both equations). The shape of the association was similar for the CKD-EPI and the FAS equation, and the increase in risk for CKD with increasing serum TSH levels was more pronounced in the range of lower serum TSH levels. We observed no significant association between serum TSH levels and albuminuria (odds ratio [OR] = 1.02; 95% confidence interval [CI]: 0.99 to 1.05; *p* = 0.090).

Individuals with high serum TSH levels had a significantly lower mean eGFR as defined by CKD-EPI and the FAS equation than individuals with TSH within the reference range. On the other hand, individuals with low serum TSH levels had a significantly higher eGFR than individuals with serum TSH levels within the reference range (Table 2). We observed no significant associations of high or low serum TSH levels with CKD as defined by CKD-EPI or the FAS equation. Likewise, high or low serum TSH levels were not significantly associated with albuminuria.

Out of the 596 individuals with low serum TSH levels, 153 individuals had increased serum fT3 or fT4 levels (25.8%) and 427 individuals had serum ft3 and ft4 levels within the reference range (72.0%). Out of the 334 individuals with high serum TSH levels, decreased serum fT3 or fT4 levels were observed in 47 individuals (14.1%) and 264 individuals had serum ft3 and ft4 levels within the reference range (79.0%). When characterizing groups of high and low TSH into subclinical and overt forms of thyroid dysfunction according to fT3 and fT4 levels, we found that individuals with subclinical but not with overt hyperthyroidism had a significantly higher adjusted mean eGFR for both equations compared to individuals with serum TSH levels within the reference range (Table 3). On the other hand, individuals with subclinical or overt hypothyroidism had a lower eGFR for both equations compared to those with serum TSH levels within the reference range. Regarding the outcome CKD, we found significant positive associations between overt hypothyroidism and eGFR using FAS, while the association between overt hyperthyroidism and eGFR using CKD-EPI barely missed statistical significance. Subclinical hyperthyroidism was associated with a lower chance for a low eGFR for the CKD-EPI but not for the FAS equation.

**Figure 4:**
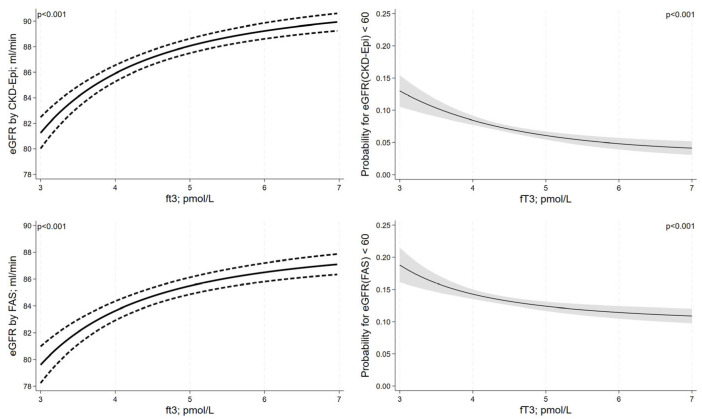
Associations between serum fT3 levels and kidney function adjusted for confounding. Results are reported as adjusted regression curves (solid line) and 95% confidence interval.

**Figure 5:**
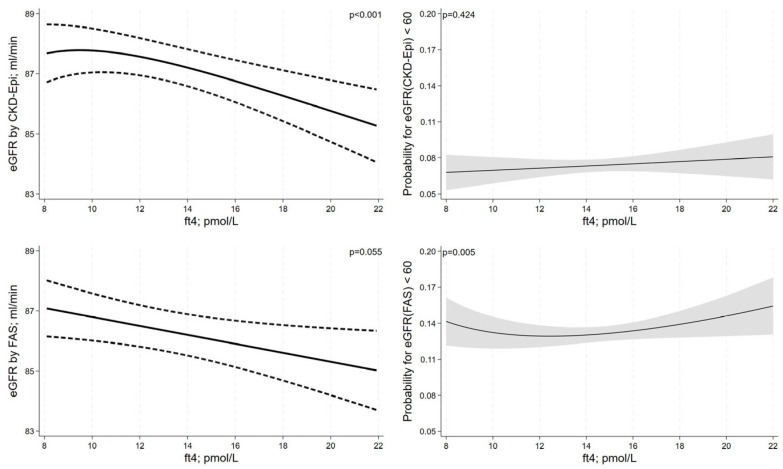
Associations between serum fT4 levels and kidney function adjusted for confounding. Results are reported as adjusted regression curves (solid line) and 95% confidence interval.

Serum fT3 levels were positively associated with eGFR as estimated using CKD-EPI or the FAS equation in a non-linear fashion (transformation: 1/fT3^2^ for both equations, Figure 4). In the lower fT3-range, there was a stronger decrease in eGFR with decreasing fT3 levels than in the upper fT3-range. Likewise, the risk for CKD with decreasing fT3 levels was more pronounced in the lower fT3-range. Serum fT4 levels were non-linearly associated with the eGFR by CKD-EPI equation showing an inverted J-shaped association (Figure 5). For the eGFR by FAS, we observed an inverse linear relationship of serum fT4 levels, which barely missed statistical significance. For the outcome CKD, we did not detect a significant association between fT4 and eGFR for the CKD-EPI equation but observed a significant U-shaped association for the FAS equation. While serum fT3 levels were not significantly associated with albuminuria (OR = 0.94; 95%-CI = 0.85 to 1.03; *p* = 0.187), we observed a positive association between serum fT4 levels and albuminuria (OR = 1.05; 95%-CI = 1.02 to 1.08; *p* = 0.003).

**Figure 6:**
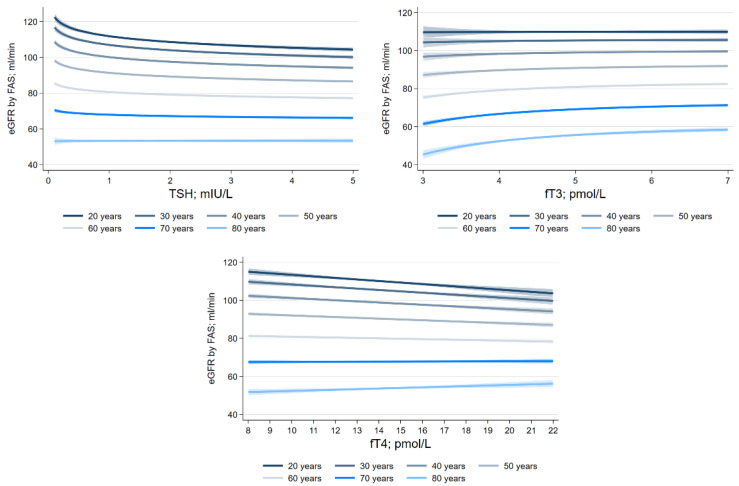
Effect modification by age on the associations between thyroid hormone levels and eGFR calculated using FAS adjusted for confounding.

We found significant interactions of TSH (*p* < 0.001), fT3 (*p* < 0.001), and fT4 (*p* < 0.001) with age for the eGFR as defined by the FAS equation (Figure 6). The inverse associations between serum TSH levels and eGFR as defined by the FAS equation were strongest in the youngest age groups, while the positive associations between serum fT3 levels and eGFR as defined by the FAS equation were strongest in older participants. The associations between serum fT4 levels and eGFR as defined by the FAS equation were inverse in the younger age groups but positive in older participants. For the CKD-EPI equation, we observed a similar significant interaction between fT4 and age (*p* = 0.017), whereas the interactions of TSH (*p* = 0.154) or fT3 (*p* = 0.351) with age were not significant. We found no significant interactions of serum TSH, fT3, or fT4 levels with sex on the eGFR outcomes.

## 4. Discussion

In pooled data of three population-based studies, we showed inverse non-linear associations of serum TSH levels with the eGFR, while serum fT3 levels were positively associated with the eGFR. High as well as low serum fT4 levels were associated with a higher risk for CKD. Furthermore, we demonstrated that age modifies the association between thyroid hormone levels and the eGFR. In contrast to the eGFR, we could not show any significant associations between thyroid hormone levels and albuminuria.

In agreement with our results, a large meta-analysis including data from 15 cross-sectional studies showed a lower eGFR in individuals with high serum TSH levels [7]. Overall, this finding argues for a higher risk for CKD in individuals with hypothyroidism even in its subclinical form. In our study, high TSH levels in combination with serum fT3 and fT4 levels in the reference range were associated with lower eGFR levels but not with an eGFR < 60 mL/min. A potential mechanism for this association between hypothyroidism and a lower eGFR may be the consequence of a reduced renal blood flow associated with hypothyroidism [19,20]. Furthermore, hypothyroidism is associated with a decreased cardiac output [19], an increased peripheral vascular resistance [21], and intrarenal vasoconstriction [22], which may lead to a decrease in overall systemic blood volume and to alterations in renal hemodynamics, resulting in a decline in the eGFR [23,24]. These mechanisms may also explain the association of high serum TSH levels within the reference range with a lower eGFR, which has been previously reported in population-based studies [5,6] and was also observed in our study.

The meta-analysis by Meuwese et al. showed cross-sectional associations of hypothyroidism with CKD but observed no significant association between hypothyroidism at baseline and changes in eGFR during follow-up [7]. Potentially, reverse causation may explain this finding, because reduced renal function can result in higher serum iodine levels [25], and an increased prevalence of subclinical and overt hypothyroidism has been observed in CKD patients [26]. In line with this, a previous study showed that iodine restriction may enhance hypothyroidism in patients with end-stage renal disease [25]. Therefore, a declining renal function might improve the thyroid hormone production through higher serum iodine levels. Potentially, thyroid hormone intake may also explain the lack of an association in the longitudinal meta-analyses [7]. Supplementation of thyroid hormones, in previously hypothyroid individuals, increases renal blood flow [20], which may result in a slower decline in renal function. However, the results remained stable in the longitudinal meta-analyses after excluding individuals with thyroid hormone intake [7]. In our analyses, we adjusted for thyroid medication intake. Furthermore, a Mendelian randomization analysis in a substantial study population showed associations of hypothyroidism and increased TSH with CKD, indicating a causal relationship between thyroid function and the kidney [8].

Even though a large number of studies already investigated associations between serum TSH levels and kidney function, not many studies examined associations between fT3 and fT4 levels and kidney function. Similar to our findings, a population-based analysis conducted in 12,785 adult individuals from the ARIC study found that higher TSH and lower fT3 levels were associated with a higher chance for an eGFR < 60 mL/min [27]. Also, the shape of the association between serum fT4 levels and eGFR < 60 mL/min in that study looked quite similar to our results. In the ARIC study, serum fT4 levels were stratified into quartiles, and individuals in the lowest and highest fT4 quartiles had the highest chance for an eGFR < 60 mL/min. In our analysis, we modeled serum fT4 levels continuously and demonstrated a U-shaped association between serum fT4 levels and an eGFR < 60 mL/min based on the FAS equation.

In interactional analyses, we could show that age modified the association between serum fT4 levels and eGFR values. In older people, lower serum fT4 levels were associated with a lower mean eGFR, which fits well with the associations found for TSH and fT3. In older people also, the association between lower fT3 levels and a lower eGFR was stronger than in younger individuals. This may point toward a more detrimental effect of low thyroid function on CKD in the elderly and that, particularly in that age group, individuals may benefit most from thyroid hormone testing to prevent future CKD events.

In contrast to our results in older individuals, lower serum fT4 levels were associated with a higher mean eGFR in younger individuals. Since the mean eGFR is much higher in younger individuals than in older people, and serum fT4 levels are also more frequently in the reference range, this finding may just be related to small differences within the fT4 reference range without any clinical meaning. Nevertheless, further studies are warranted to investigate the relationship between serum fT4 levels and reduced kidney function.

In our analyses, associations of thyroid function with eGFR were stronger for the FAS compared to the CKD-EPI equation. Previous studies suggested that the FAS equation is superior to the CKD-EPI equation in older subjects as it better accounts for the fostered physiological decline in eGFR observed in individuals older than 40 years [16,28]. Indeed, the number of individuals with an eGFR < 60 mL/min/1.73 m^2^ was higher for the FAS than for the CKD-EPI equation in our study. In line with this, a study reporting data from BASE-II, which is also part of the present analyses, concluded that the FAS equation should be used in older subjects [29], while another study found no significant differences when comparing the two equations. In our study population with a median age of 55 years, we believe that the FAS equation is more appropriate than the CKD-EPI equation [30].

In our study we found a positive association between serum fT4 levels and albuminuria, but no such association for TSH or fT3. The literature showed associations of subclinical hypothyroidism [9] or low fT3 levels [10] with albuminuria, which is in contrast to our findings. The difference in results might be related to different ethnicities, because the two latter studies were both conducted in Asian populations [9,10]. Furthermore, one of those studies was conducted in health-check-up individuals [9], which may represent only a relatively healthy snapshot of the general Japanese population. In the other study, results were only reported for fT3 but not for TSH or fT4 [10]. Even in individuals with advanced CKD, no significant association between thyroid hormone status and albuminuria was observed [31]. Overall, findings regarding the association of thyroid hormone levels with albuminuria in the general population are inconsistent and require further investigation. However, in light of the available literature, it seems that thyroid function is more strongly associated with the eGFR than with albuminuria.

A limitation in the SHIP population is that about 25% of the urinary albumin measurements were below the detection limit of 5 mg/L, allowing only usage of the dichotomized albuminuria variable. A further limitation is the lack of a second eGFR measurement three months after the initial one, which hampered the definition of CKD. Furthermore, reverse causation cannot be ruled out, since our analyses only included cross-sectional data. Another limitation is that the GFR was only estimated, but we included two commonly used equations for GFR estimation in our analyses. The strengths of our study are the inclusion of three large population-based studies with a broad age range and the availability of TSH, fT3, and fT4 as markers of thyroid function. We used fractional polynomials to determine non-linear relationships between markers of thyroid and kidney function.

In conclusion, we showed that high TSH, low fT3, as well as low and high fT4 levels were independently associated with a reduced kidney function in a non-linear fashion. Our results indicate that high TSH/hypothyroidism might be associated with a reduced kidney function. Thyroid function might be more tightly related to the eGFR than to albuminuria in the general population.

## Figures and Tables

**Table 1:** Characteristics of the study population stratified by serum TSH levels.

	TSH in Reference Range (n = 7003)	Low TSH (n = 596)	High TSH (n = 334)
**Study**			
SHIP-START-2	26.2%	37.6%	18.0%
SHIP-TREND-0	50.1%	58.4%	45.5%
BASE-II	23.7%	4.0%	36.5%
**Age; years**	54.6 (15.7)	60.7 (13.2)	51.8 (17.7)
**Females**	50.3%	54.0%	63.5%
**Smoking status**			
Never	39.8%	36.7%	40.7%
Former	37.7%	43.1%	34.1%
Current	22.6%	20.1%	25.2%
**Body mass index; kg/m^2^**	27.6 (5.0)	28.3 (5.1)	27.2 (5.7)
**Waist circumference; cm**	92 (14)	93 (14)	90 (15)
**Hypertension**	48.9%	61.1%	39.4%
**Type 2 diabetes**	9.5%	13.8%	8.4%
**HDL-cholesterol; mmol/L**	1.47 (0.40)	1.45 (0.39)	1.53 (0.43)
**LDL-cholesterol; mmol/L**	3.31 (0.95)	3.36 (0.98)	3.30 (1.03)
**TSH; mIU/L**	1.45 (0.72)	0.31 (0.14)	6.51 (7.82)
**fT3; pmol/L**	4.73 (0.88)	4.78 (0.99)	4.69 (0.95)
**fT4; pmol/L**	14.0 (2.3)	15.1 (3.2)	13.6 (3.0)
**Thyroid medication**	9.7%	25.7%	24.3%
**Serum creatinine; µmol/L**	78 (17)	77 (19)	79 (17)
**eGFR (CKD-EPI); mL/min**	87 (18)	84 (19)	86 (19)
**eGFR (CKD-EPI) < 60 mL/min**	6.9%	11.7%	8.4%
**eGFR (FAS); mL/min**	84 (22)	80 (23)	81 (21)
**eGFR (FAS) < 60 mL/min**	12.7%	19.6%	14.7%
**Urea; mmol/L**	5.1 (1.5)	5.4 (1.9)	5.0 (1.4)
**Uric acid; µmol/L**	294 (81)	292 (82)	285 (81)
**ACR ≥ 30 mg/g**	11.9%	15.8%	14.1%

TSH reference limits according to [17]; ACR, urinary albumin–creatinine ratio. Data are expressed as mean and standard deviation for continuous variables and as percentage for categorical variables.

**Table 2:** Associations of low and high TSH levels with markers of renal function.

	TSH < 0.49 mIU/L ^#^ β (95%-CI)	TSH ≥ 3.29 mIU/L ^#^ β (95%-CI)
**eGFR (CKD-EPI); mL/min**	2.26 (1.14; 3.83) *	−3.71 (−5.16; −2.25) *
**eGFR (FAS); mL/min**	2.44 (1.16; 3.71) *	−4.14 (−5.78; −2.49) *
	**Odds ratio (95%-CI)**	**Odds ratio (95%-CI)**
**eGFR (CKD-EPI) < 60 mL/min**	0.88 (0.65; 1.20)	1.30 (0.82; 2.05)
**eGFR (FAS) < 60 mL/min**	0.78 (0.59; 1.03)	1.26 (0.84; 1.88)
**ACR ≥ 30 mg/g**	0.96 (0.75; 1.22)	1.35 (0.97; 1.88)

^#^ compared to TSH in the range; CI, confidence interval; * *p* < 0.05. Estimates are derived from linear (continuous outcomes) and logistic regression (dichotomous outcomes) models adjusted for age, sex, smoking status, intake of thyroid medication, and study.

**Table 3:** Associations of thyroid function with markers of renal function.

	Subclinical Hyper vs. Euthyroid β (95%-CI)	Overt Hyper vs. Euthyroid β (95%-CI)	Subclinical Hypo vs. Euthyroid β (95%-CI)	Overt Hypo vs. Euthyroid β (95%-CI)
eGFR (CKD-EPI); mL/min	2.86 (1.56; 4.16) *	0.92 (−1.24; 3.08)	−2.85 (−4.47; −1.23) *	−7.98 (−11.76; −4.19) *
eGFR (FAS); mL/min	3.04 (1.57; 4.51) *	1.02 (−1.43; 3.47)	−3.35 (−5.19; −1.51) *	−7.33 (−11.62; −3.03) *
	Odds ratio (95%-CI)	Odds ratio (95%-CI)	Odds ratio (95%-CI)	Odds ratio (95%-CI)
eGFR (CKD-EPI) < 60 mL/min	0.67 (0.45; 0.99) *	1.36 (0.82; 2.27)	0.94 (0.52; 1.67)	2.34 (0.99; 5.56)
eGFR (FAS) < 60 mL/min	0.78 (0.567; 1.09)	0.78 (0.47; 1.28)	0.91 (0.56; 1.48)	2.65 (1.15; 6.07) *
ACR ≥ 30 mg/g	0.94 (0.71; 1.25)	1.17 (0.77; 1.80)	1.35 (0.93; 1.97)	1.34 (0.63; 2.84)

Compared to TSH in the range; CI, confidence interval; * *p* < 0.05. Estimates are derived from linear (continuous outcomes) and logistic regression (dichotomous outcomes) models adjusted for age, sex, smoking status, intake of thyroid medication, and study. Participants were categorized according to thyroid function status: **subclinical hyper (n = 427):** low tsh, ft3, and ft4 in the reference range; **overt hyper (n = 153):** low tsh, ft3, or ft4 increased; **subclinical hypo (n = 264):** high tsh, ft3, and ft4 in the reference range; **overt hypo (n = 47):** high tsh, ft3, or ft4 decreased; **euthyroid:** TSH in the reference range.

## Data Availability

Data from the “Study of Health of Pomerania” are available from the University Medicine Greifswald, Germany, but restrictions apply to the availability of these data, which were used under license for the current study, and so are not publicly available. Data are, however, available upon reasonable request at https://transfer.ship-med.uni-greifswald.de/FAIRequest/ (accessed on 31 August 2023) and with permission of the University Medicine Greifswald.

## References

[1] Kidney Disease: Improving Global Outcomes (KDIGO) CKD Work Group (2013). KDIGO clinical practice guideline for the evaluation and management of chronic kidney disease. Kidney Int. Suppl..

[2] Chronic Kidney Disease Prognosis C., Matsushita K., van der Velde M., Astor B.C., Woodward M., Levey A.S., de Jong P.E., Coresh J., Gansevoort R.T. (2010). Association of estimated glomerular filtration rate and albuminuria with all-cause and cardiovascular mortality in general population cohorts: A collaborative meta-analysis. Lancet.

[3] Hillege H.L., Fidler V., Diercks G.F., van Gilst W.H., de Zeeuw D., van Veldhuisen D.J., Gans R.O., Janssen W.M., Grobbee D.E., de Jong P.E. (2002). Urinary albumin excretion predicts cardiovascular and noncardiovascular mortality in general population. Circulation.

[4] Lee M., Saver J.L., Chang K.H., Liao H.W., Chang S.C., Ovbiagele B. (2010). Impact of microalbuminuria on incident stroke: A meta-analysis. Stroke.

[5] Asvold B.O., Bjoro T., Vatten L.J. (2011). Association of thyroid function with estimated glomerular filtration rate in a population-based study: The HUNT study. Eur. J. Endocrinol..

[6] Gopinath B., Harris D.C., Wall J.R., Kifley A., Mitchell P. (2013). Relationship between thyroid dysfunction and chronic kidney disease in community-dwelling older adults. Maturitas.

[7] Meuwese C.L., van Diepen M., Cappola A.R., Sarnak M.J., Shlipak M.G., Bauer D.C., Fried L.P., Iacoviello M., Vaes B., Degryse J. (2019). Low thyroid function is not associated with an accelerated deterioration in renal function. Nephrol. Dial. Transplant..

[8] Ellervik C., Mora S., Ridker P.M., Chasman D.I. (2020). Hypothyroidism and Kidney Function: A Mendelian Randomization Study. Thyroid.

[9] Toda A., Hara S., Tsuji H., Arase Y. (2020). Subclinical hypothyroidism is associated with albuminuria in Japanese nondiabetic subjects. Endocrine.

[10] Zhou Y., Ye L., Wang T., Hong J., Bi Y., Zhang J., Xu B., Sun J., Huang X., Xu M. (2014). Free triiodothyronine concentrations are inversely associated with microalbuminuria. Int. J. Endocrinol..

[11] El-Eshmawy M.M., Abd El-Hafez H.A., El Shabrawy W.O., Abdel Aal I.A. (2013). Subclinical hypothyroidism is independently associated with microalbuminuria in a cohort of prediabetic egyptian adults. Diabetes Metab. J..

[12] Volzke H., Schossow J., Schmidt C.O., Jurgens C., Richter A., Werner A., Werner N., Radke D., Teumer A., Ittermann T. (2022). Cohort Profile Update: The Study of Health in Pomerania (SHIP). Int. J. Epidemiol..

[13] Bertram L., Bockenhoff A., Demuth I., Duzel S., Eckardt R., Li S.C., Lindenberger U., Pawelec G., Siedler T., Wagner G.G. (2014). Cohort profile: The Berlin Aging Study II (BASE-II). Int. J. Epidemiol..

[14] Gerstorf D., Bertram L., Lindenberger U., Pawelec G., Demuth I., Steinhagen-Thiessen E., Wagner G.G. (2016). Editorial. Gerontology.

[15] Stevens L.A., Padala S., Levey A.S. (2010). Advances in glomerular filtration rate-estimating equations. Curr. Opin. Nephrol. Hypertens..

[16] Pottel H., Hoste L., Dubourg L., Ebert N., Schaeffner E., Eriksen B.O., Melsom T., Lamb E.J., Rule A.D., Turner S.T. (2016). An estimated glomerular filtration rate equation for the full age spectrum. Nephrol. Dial. Transplant..

[17] Ittermann T., Khattak R.M., Nauck M., Cordova C.M., Volzke H. (2015). Shift of the TSH reference range with improved iodine supply in Northeast Germany. Eur. J. Endocrinol..

[18] Schmidt C.O., Ittermann T., Schulz A., Grabe H.J., Baumeister S.E. (2013). Linear, nonlinear or categorical: How to treat complex associations in regression analyses? Polynomial transformations and fractional polynomials. Int. J. Public Health.

[19] Crowley W.F., Ridgway E.C., Bough E.W., Francis G.S., Daniels G.H., Kourides I.A., Myers G.S., Maloof F. (1977). Noninvasive evaluation of cardiac function in hypothyroidism. Response to gradual thyroxine replacement. N. Engl. J. Med..

[20] Villabona C., Sahun M., Roca M., Mora J., Gomez N., Gomez J.M., Puchal R., Soler J. (1999). Blood volumes and renal function in overt and subclinical primary hypothyroidism. Am. J. Med. Sci..

[21] Diekman M.J., Harms M.P., Endert E., Wieling W., Wiersinga W.M. (2001). Endocrine factors related to changes in total peripheral vascular resistance after treatment of thyrotoxic and hypothyroid patients. Eur. J. Endocrinol..

[22] Singer M.A. (2001). Of mice and men and elephants: Metabolic rate sets glomerular filtration rate. Am. J. Kidney Dis..

[23] Chonchol M., Lippi G., Salvagno G., Zoppini G., Muggeo M., Targher G. (2008). Prevalence of subclinical hypothyroidism in patients with chronic kidney disease. Clin. J. Am. Soc. Nephrol..

[24] Toft A.D., Boon N.A. (2000). Thyroid disease and the heart. Heart.

[25] Iglesias P., Bajo M.A., Selgas R., Diez J.J. (2017). Thyroid dysfunction and kidney disease: An update. Rev. Endocr. Metab. Disord..

[26] Lo J.C., Chertow G.M., Go A.S., Hsu C.Y. (2005). Increased prevalence of subclinical and clinical hypothyroidism in persons with chronic kidney disease. Kidney Int..

[27] Schultheiss U.T., Daya N., Grams M.E., Seufert J., Steffes M., Coresh J., Selvin E., Kottgen A. (2017). Thyroid function, reduced kidney function and incident chronic kidney disease in a community-based population: The Atherosclerosis Risk in Communities study. Nephrol. Dial. Transplant..

[28] Lengnan X., Aiqun C., Ying S., Chuanbao L., Yonghui M. (2021). The effects of aging on the renal function of a healthy population in Beijing and an evaluation of a range of estimation equations for glomerular filtration rate. Aging.

[29] Konig M., Gollasch M., Demuth I., Steinhagen-Thiessen E. (2017). Prevalence of Impaired Kidney Function in the German Elderly: Results from the Berlin Aging Study II (BASE-II). Gerontology.

[30] Da Silva Selistre L., Rech D.L., de Souza V., Iwaz J., Lemoine S., Dubourg L. (2019). Diagnostic Performance of Creatinine-Based Equations for Estimating Glomerular Filtration Rate in Adults 65 Years and Older. JAMA Intern. Med..

[31] Reinhardt W., Mulling N., Behrendt S., Benson S., Dolff S., Fuhrer D., Tan S. (2021). Association between albuminuria and thyroid function in patients with chronic kidney disease. Endocrine.

